# Dose-dependent effects of intravenous methoxamine infusion during hip-joint replacement surgery on postoperative cognitive dysfunction and blood TNF-α level in elderly patients: a randomized controlled trial

**DOI:** 10.1186/s12871-017-0367-6

**Published:** 2017-06-09

**Authors:** Shenghui Sun, Defeng Sun, Lin Yang, Jun Han, Ruochuan Liu, Lijie Wang

**Affiliations:** 1 Affiliated High School of Liaoning Normal University, Dalian, People’s Republic of China; 2 Department of Anesthesiology, The First Affiliated Hospital of Dalian Medical University, No.222 ZhongshanRoad, Xigang District, Dalian, 116011 Liaoning People’s Republic of China; 3 Department of Neuroelectrophysiology, The First Affiliated Hospital of Dalian Medical University, Dalian, People’s Republic of China

**Keywords:** Methoxamine, Elderly patients, Hip-joint replacement surgery, Postoperative cognitive dysfunction, Tumor necrosis factor-α

## Abstract

**Background:**

Postoperative cognitive dysfunction (POCD), common in elderly patients, is thought to be closely associated with intraoperative instability of hemodynamics and excessive excretion of tumor necrosis factor-α (TNF-α). Methoxamine is a blood-pressure increasing drug commonly used for maintaining intraoperative hemodynamics. Methoxamine potentially promotes TNF-α expression, leading to an increased risk of POCD. This study aimed to investigate the dose-dependent effect of methoxamine on the incidence of early POCD and blood TNF-α level.

**Methods:**

This single-center prospective double-blind controlled clinical trial included a total of 300 adult patients (75–90 years old, American Society of Anesthesiologists class II–III) who underwent unilateral hip-joint replacement surgery under epidural anesthesia. Patients were randomly divided into three methoxamine groups (M1, M2, and M3), and one control group (*n* = 75 per group). During surgery, M1, M2, and M3 patients received intravenous infusion of methoxamine at 2, 3, or 4 μg·kg^−1^·min^−1^, respectively; the control group received saline of same volume at the same infusion rate. All patients received standard transfusion to maintain stable circulation. Hemodynamics, cardiovascular events, and serum TNF-α levels were monitored. Mini Mental State Examination was performed both before and after surgery to diagnose POCD.

**Results:**

The primary outcome of this study was the incidence of POCD, which was higher in the M3 group (18.7%) than in the control group (5.3%), the M1 group (6.7%), or the M2 group (6.7%) (all *P* < 0.05). The secondary outcomes were the postoperative blood TNF-α level and intraoperative hemodynamic parameters. The postoperative TNF-α level was found to be higher than baseline in all groups and was highest in M3 patients (*P* < 0.05). The intraoperative hemodynamic parameters showed improved stability in the M1 and M2 groups compared with the control group. However, in the M3 group, abnormally increased intraoperative blood pressure, cardiac output, and systolic stroke volume were observed.

**Conclusions:**

Intravenous infusion of methoxamine at 2–3 μg·kg^−1^·min^−1^ can maintain stable hemodynamics in elderly patients during epidural anesthesia for hip-joint replacement surgery, without increasing the incidence of POCD. Increasing the dose to 4 μg·kg^−1^·min^−1^ provided no further advantages but induced adverse effects on the intraoperative hemodynamics.

**Trial registration:**

Chinese Clinical Trial Register (Unique identifier: ChiCTR-INR-15007607, retrospectively registered 18 Dec 2015).

## Background

Postoperative cognitive dysfunction (POCD), common in the elderly, refers to a new cognitive impairment after surgery, especially decline in memory and executive functions [1]. POCD can lead to personality changes, social skill deficits, and emotional problems including anxiety [2–4]. POCD can significantly interfere with postoperative recovery, increasing patients’ morbidity and mortality rates as well as hospital expenses [5, 6]. It often lasts from a few days to a few weeks after surgery, but has the potential to persist for or occur after several weeks to 7.5 years [7].

Although it is still unclear at present, the mechanism of POCD is considered to involve dysfunction of the central nervous, endocrine, and immune systems, caused by multiple factors associated with surgery and anesthesia [8]. The peripheral immune system can be activated by surgical injury, leading to the release of various inflammatory cytokines including tumor necrosis factor-alpha (TNF-α), which can diffuse through the blood–brain barrier and cause cognitive deficits [9, 10]. Furthermore, it has been demonstrated that beta-adrenoceptor activation suppresses TNF-α release, whereas alpha-adrenoceptor activation stimulates TNF-α release [11, 12].

Because epidural anesthesia is thought to be associated with a relatively low incidence of POCD, it is commonly used for hip-joint replacement surgery [13]. Elderly patients are the major recipients of hip-joint replacement. In patients older than 70 years, who often have compromised critical function and are highly sensitive to drugs, epidural anesthesia and the surgical procedure can induce significant physiological changes including disturbance of hemodynamics, hypotension, and bradycardia, especially in patients with high blood pressure and coronary artery disease [14, 15]. Therefore, vasoactive drugs, including ephedrine and dopamine, are often used clinically to maintain stable hemodynamics during hip-joint replacement surgeries, which is important for the balance between oxygen supply and consumption in the myocardium and the prevention of pulmonary edema caused by fast or excessive fluid transfusion. However, ephedrine and dopamine can also increase the heart rate and myocardium oxygen consumption while increasing the blood pressure and therefore are commonly substituted with methoxamine in current practice [16, 17].

Methoxamine is a sympathomimetic drug that can directly activate peripheral vascular α_1_-adrenergic receptors, leading to vessel constriction and increases in both systolic and diastolic blood pressure. These increased blood pressures can further activate the carotid sinus baroreflex, decreasing the heart rate and myocardium oxygen consumption. Furthermore, methoxamine itself does not alter myocardium excitability or increase myocardium oxygen consumption even as it increases the blood flow in the coronary artery and endocardium [17, 18]. Muscular or intravascular bolus injection of methoxamine often fails to maintain stable hemodynamics because of a delayed onset and short duration of action. In a previous study, we demonstrated that continuous intravenous infusion of methoxamine (2 μg·kg^−1^·min^−1^) can provide safe and effective maintenance of hemodynamics under epidural anesthesia during hip-joint replacement surgery in elderly patients [19].

In summary, POCD is thought to be closely associated with intraoperative instability of hemodynamics and excessive excretion of TNF-α [20]. Methoxamine is a blood-pressure increasing drug commonly used for maintaining intraoperative hemodynamics. Methoxamine potentially promotes TNF-α expression, leading to an increased risk of POCD. The primary aim of his study was to investigate the effect of intraoperative infusion of methoxamine at three different dosages of methoxamine on the incidence of early POCD and blood TNF-α level. The secondary aim of this study was to provide evidence for choosing the optimal methoxamine dosage that can maintain stable hemodynamics during surgery without increasing the risk of early POCD.

In the present study, we aimed to investigate whether improved management of hemodynamics through appropriate methoxamine administration could lead to a lower incidence of early POCD and observe how it affects blood TNF-α levels. Three different dosages of methoxamine were investigated. The results were expected to provide evidence for choosing the optimal methoxamine dosage that can maintain stable hemodynamics during surgery without increasing the risk of early POCD.

## Methods

### Ethics and patients

This study was preapproved by our institutional Research Ethics Committee in 2012 (Identification No.: KY2012–42) and then retrospectively registered with the internationally recognized Chinese Ethics Committee of Registering Clinical Trials in 2015 (Unique identifier: ChiCTR-INR-15007607). Written informed consent was obtained from each patient or his/her authorized representative(s) when he/she was in a critical physical condition or mentally challenged. Between January 1, 2013 and September 30, 2015, 141 male and 159 female patients (*n* = 300) underwent unilateral hip-joint replacement surgery under epidural anesthesia in our Hospital.

Inclusion criteria: (1) patient was aged between 75 and 90 years old; (2) patient had a physical status classification of American Society of Anesthesiologists (ASA) II or III (i.e., mild but definite systemic disease such as mild diabetes and chronic sinusitis, or severe systemic disease including severe diabetes and severe trauma); (3) patient was scheduled for unilateral hip-joint replacement surgery under epidural anesthesia.

Exclusion criteria: (1) Mini Mental State Examination (MMSE) score of <23 points; (2) obvious liver or kidney dysfunction; (4) history of mental illnesses or neurological diseases; (5) history of taking tranquilizers or antidepressants, or history of alcohol abuse; (6) serious audio-visual obstacles that affected communication; (7) an education level lower than junior high school; (8) blood electrolyte imbalance or hyperglycemia; (9) severe vision or hearing loss; and (10) recent treatments with sedatives, antidepressants, analgesics, or monoamine oxidase inhibitors.

### Interventions

Patients were randomly assigned to four groups based on assignments found in the numbered envelopes, as follows: a control group receiving intravenous saline infusion (Group C, *n* = 75) and three methoxamine groups receiving 2, 3, or 4 μg·kg^−1^·min^−1^ (10 mL/h) methoxamine (i.e., Groups M1, M2, and M3, *n* = 75 in each group). Patients in the control group received an infusion of normal saline (10 mL/h).

### Epidural anesthesia procedures and hemodynamic maintenance

Patients were subjected to 8 h food deprivation and 2 h water deprivation before surgery, without preoperative treatment. Epidural anesthesia was performed by injecting 2% lidocaine (3 mL per patient) into the epidural space via a catheter secured at the L2–L3 vertebral interval while the patient was in a side-lying position. The radial artery was catheterized for real-time blood pressure monitoring. After the level of anesthesia reached the T6–T8 vertebral level, 1% ropivacaine (5–10 mL per patient) was injected into the same catheter at the L2–L3 vertebral interval. The M1, M2, and M3 groups received an intravenous infusion of methoxamine (catalogue number: 120,901, Grand Pharma (China) Co. Ltd., Wuhan) at dosages of 2, 3, and 4 μg·kg^−1^·min^−1^ (10 mL/h) respectively, whereas patients in the control group were infused with normal saline (10 mL/h). To maintain stable circulation during surgery, sodium lactate Ringer’s solution and 6% hydroxyethyl starch 130/0.4 in 0.9% sodium chloride (Voluven®; Fresenius KabiNorge A.S., Halden, Norway) were administered intravenously first at a rate of 0.5–1.0 mL·kg^−1^·min^−1^ for 20–40 min and then maintained at 0.25 mL·kg^−1^·min^−1^ with a crystal colloid ratio of 3:1. If the intraoperative blood pressure was lower than 70% of the baseline blood pressure (the reading at 10 min before anesthesia), the infusion rate was increased or ephedrine (5–6 mg) was administered intravenously. Depending on the surgical practice, 3–5 mL of 1% ropivacaine was added per patient as needed.

### Physiological monitoring during anesthesia

During anesthesia preparation and surgery, patients were monitored by electrocardiogram and measurement of mean arterial blood pressure (MAP), central venous pressure (CVP), heart rate (HR), rate pressure product (RPP) (HR × systolic blood pressure), saturation of peripheral oxygen (SpO_2_), cardiac output (CO), and stroke volume (SV), using a Beneview T8 patient monitor (Shenzhen Mindray Bio-Medical Electronics Co., Ltd., Shenzhen, China) and the FloTrac Vigileo Sensor (Edwards Lifesciences LLC, USA). Values for these parameters were noted at 10 min before anesthesia (T1); 10 min (T2), 30 min (T3), and 1 h (T4) after anesthesia; and at the conclusion of surgery (T5). The radial artery and internal jugular vein were catheterized for intravascular monitoring of MAP and CVP. Low flow high-concentration oxygen (1–2 L/min) was delivered via face mask, resulting in an inspired oxygen concentration of 25%–29%. Blood samples were collected from the nontransfused forearm (3 mL per patient) at 10 min before anesthesia, at the end of surgery, and at 1 and 2 days after surgery. The serum TNF-α level was measured using an enzyme-linked immunosorbent assay (ELISA). The total volume of liquid transfused, the dosage of ephedrine delivered during surgery, and the dosages of ropivacaine and sufentanil during and after surgery were noted. Urine volumes during surgery and 24 h after surgery were quantified. The incidences of pulmonary edema, high blood pressure (30% higher than that at baseline), low blood pressure (<70% of that at baseline), tachycardia (HR > 100 beats/min), and bradycardia (HR <60 beats/min) during surgery were counted, and the accumulated duration of low blood pressure was calculated. In cases of either high or low blood pressure, patients were immediately treated with either continuous intravenous nitroglycerin infusion at 1–3 μg·kg^−1^·min^−1^ or an increased transfusion rate and intravenous injection of ephedrine (5–6 mg per patient), respectively. Tachycardia and bradycardia were treated with immediate intravenous injection of esmolol hydrochloride (10–20 mg) or atropine (0.5–1.0 mg), respectively. When the SpO_2_ was lower than 90%, the oxygen pressure in the inspired air was increased.

### Use of analgesics

All patients received sufentanil and ropivacaine for pain control after surgery. In each patient, sufentanil (30 mcg) mixed with 0.2% ropivacaine and diluted in a total volume of 100 mL was administered via a pump (Royal Fornia Medical, Zhuhai, China) for patient-controlled epidural analgesia (PCEA) for 48 h. The pump was programmed to deliver drugs in a 0.5-mL bolus with a lockout time of 15 min and a background infusion rate of 2 mL/h [21].

### Assessment of postoperative analgesia quality

Analgesia quality and comfort level were assessed at 1, 6, 12, 18, 24, 36, and 48 h postoperation. The analgesia quality was assessed using the visual analogue scale (VAS) with 0 for no pain, 10 points for drastic pain, >5 points for unsatisfactory analgesia, 3–4 points for acceptable analgesia, and <3 points for satisfactory analgesia [22]. The comfort level was evaluated using the Bruggrmann Comfort Scale (BCS) with 0 for persistent pain, 1 for severe pain while moving the operated limb, 2 for mild pain while moving the operated limb, 3 for no pain while moving the operated limb, and 4 for no pain while slightly touching the incision [23].

### Assessment of cognitive function

The MMSE has been extensively used in clinical and research settings for assessing cognitive impairment. It is a simple, practical, and highly reliable 30-point questionnaire that excludes the influences of emotional and mental disorders on cognitive function [24]. The MMSE test was conducted in each patient 1 day before and 1, 3, and 7 days after surgery by the same psychologist. The functions that were examined included orientation (temporal orientation, 5 points; spatial orientation, 5 points), recall (immediate recall, 3 points; delayed recall, 3 points), language (articulation, 2 points; recitation, 1 point; writing, 1 point), execution (ability to follow simple commands, 1 point), and calculation (5 points). POCD was defined as decreases in MMSE test scores by more than 2 points from scores before surgery [24].

### Measurement of blood TNF-α level

Blood samples were collected from the nontransfused forearm (3 mL per patient) at 10 min before anesthesia, at the end of surgery, and at 1 and 2 days after surgery on an empty stomach. After collection, the samples were immediately dissolved in 5 mL ethylene diamine tetraacetic acid. The solution was then centrifuged in a Beckman Allegra TM21 R centrifuge at 3000 r/min for 5 min. Then the supernatant serum was separated and stored at 4°∁. An ELISA kit (Shanghai Xinyu Biology, Ltd) was used to measure the TNF-α concentration.

### Sample size estimation and statistical analysis

The sample size was estimated for detecting differences in the primary outcome variable (i.e., incidence of POCD) using Power Analysis and Sample Size 11.0 (PASS 11.0) with 90% power (1–β) and α = 0.05 (two-sided). According to and results from a pilot study, the incidence rates of POCD were found to be 4%, 10%, 15%, and 25% in a control group and low-dose, moderate-dose, and high-dose methoxamine groups, respectively. The effect size, i.e., the minimum detectable difference among the four groups, was calculated to be 0.225 based on a contingency table [20], and the minimum sample size was found to be 280 for all four groups (*n* = 70 in each group) according to Chi-square test for multiple proportions. Considering some participants might drop out, the final sample size was set at *n* = 75 for each group.

Statistical analyses of inter-group differences were performed using SPSS 15.0 (SPSS Inc., Chicago, IL, USA). All numerical values are presented as mean ± standard deviation (SD). Repeated measures ANOVA were used to compare intra-group differences. One-way ANOVA and post-hoc tests were performed to compare inter-group differences. Categorical data were compared using the chi-square test. Statistical significance was accepted at *P* < 0.05.

## Results

The incidence of POCD was the primary outcome of this study. The general clinical information, operation time, and red blood cell count for the four study groups are presented in Table 1. There were no statistical differences in any of the parameters among the groups (*P* > 0.05).

**Table 1:** General clinical parameters, operation time, and perioperative red blood cell count in four groups (*n* = 75 per group)

Group	Male/female, n/n	Age, years	Body weight, kg	HBP, *n* (%)	Cardiac disease, *n* (%)	Pulmonary disease, *n* (%)	Duration of anesthesia, min	Perioperative RBC transfusion volume, mL	Highest education level (JHS/HS/college or higher), n/n/n
C	35/40	82.5 ± 7.5	72.9 ± 14.6	16 (21.3)	25 (33.3)	19 (25.3)	157.9 ± 18.7	127.3 ± 26.2	17/30/28
M1	34/41	82.5 ± 7.0	72.7 ± 14.2	16(21.3)	26 (34.7)	20 (26.7)	159.8 ± 17.6	126.8 ± 24.5	15/31/29
M2	37/38	83.5 ± 6.5	72.5 ± 15.5	19 (25.3)	24 (32.0)	18 (24.0)	161.1 ± 16.5	134.3 ± 30.4	18/28/29
M3	35/40	83.0 ± 7.0	73.5 ± 13.5	17 (22.7)	25 (33.3)	20 (26.7)	158.7 ± 19.3	131.5 ± 28.2	16/29/30

*Abbreviations*: *HBP* high blood pressure, *RBC* red blood cell, *JHS*, junior high school, *HS* high school

Table 2 summarizes the hemodynamic parameters at different time points in the four groups. As compared with those measured 10 min before anesthesia (T1), the control group showed decreased MAP, CVP, HR, RPP, CO, and SV; all methoxamine treatment groups (M1, M2, and M3) showed a decreased HR; the M1 and M2 groups showed a decreased CVP at all time points (T2, T3, and T4) after anesthesia and at the end of surgery (T5); and M3 group showed increased MAP, RPP, CO, and SV at T4 and T5 (*P* < 0.05). As compared with the control group, all methoxamine treatment groups had higher MAP, CVP, RPP, CO, and SV values at T2, T3, T4, and T5 (all *P* < 0.05). At T4 and T5, the M3 group had even higher MAP, CVP, RPP, CO, and SV values than the M1 and M2 groups (all *P* < 0.05).

**Table 2:** Hemodynamic parameters at different time points in four groups (*n* = 75 per group)

	C	M1	M2	M3
Mean arterial pressure (MAP) (mmHg)				
Baseline	89.7 ± 9.2	91.8 ± 9.3	91.5 ± 9.1	90.7 ± 8.9
10 min_Anesthesia	69.7 ± 6.1^a^	87.5 ± 8.5^c^	90.5 ± 9.0^c^	92.8 ± 9.1^c^
30 min_Anesthesia	71.5 ± 6.7^a^	88.1 ± 9.3^c^	92.8 ± 9.3^c^	94.4 ± 9.2^c^
60 min_Anesthesia	72.8 ± 7.4^a^	90.2 ± 9.5^c^	97.7 ± 9.6^c^	111.9 ± 13.4^acef^
End of operation	75.9 ± 8.1^a^	93.3 ± 10.3^c^	98.3 ± 10.5^c^	115.7 ± 13.6^acef^
Heart rate (HR) (beats/min)				
Baseline	81.5 ± 8.3	81.3 ± 8.4	82.3 ± 8.1	82.2 ± 8.2
10 min_Anesthesia	67.5 ± 6.2^a^	65.7 ± 7.1 ^a^	66.5 ± 7.4^a^	68.8 ± 7.8^a^
30 min_Anesthesia	69.7 ± 8.2^a^	66.9 ± 8.7 ^a^	66.6 ± 8.9^a^	67.9 ± 8.7^a^
60 min_Anesthesia	71.3 ± 8.4^a^	68.5 ± 8.9 ^a^	68.8 ± 8.7^a^	71.5 ± 8.9^a^
End of operation	71.5 ± 8.6^a^	71.5 ± 8.5 ^a^	71.3 ± 8.9^a^	71.6 ± 9.1^a^
Rate pressure product (RPP)				
Baseline	10,451.3 ± 269.5	10,487.6 ± 281.7	10,490.2 ± 290.8	10,498.6 ± 297.3
10 min_Anesthesia	7572.7 ± 187.6^a^	9695.4 ± 295.4^c^	9605.6 ± 301^c^	9612.9 ± 308.5^c^
30 min_Anesthesia	7691.3 ± 279.4^a^	9595.5 ± 297.3^c^	9609.8 ± 306.5^c^	9925.7 ± 315.7^c^
60 min_Anesthesia	7718.6 ± 286.2^a^	9601.7 ± 305.7^c^	10,313.5 ± 287.3^c^	13,076.5 ± 356.2^acef^
End of operation	7995.4 ± 297.3^a^	9611.8 ± 308.9^c^	10,482.7 ± 291.5^c^	13,218.6 ± 395.8^acef^
Central venous pressure (CVP) (cmH_2_O)				
Baseline	8.6 ± 1.2	8.7 ± 1.2	8.5 ± 1.3	8.6 ± 1.3
10 min_Anesthesia	3.9 ± 1.1^a^	5.7 ± 1.0^ac^	5.8 ± 1.1^ac^	6.2 ± 1.1^ac^
30 min_Anesthesia	4.0 ± 1.1^a^	6.0 ± 1.1^ac^	6.1 ± 1.0^ac^	6.4 ± 1.1^ac^
60 min_Anesthesia	4.5 ± 1.0^a^	6.1 ± 1.0^ac^	6.3 ± 1.1^ac^	8.5 ± 1.2^cef^
End of operation	4.7 ± 1.1^a^	6.3 ± 1.1^ac^	6.5 ± 1.0^ac^	8.7 ± 1.3^cef^
Saturation of peripheral oxygen (SpO_2_) (%)				
Baseline	98.6 ± 1.1	98.7 ± 1.1	98.8 ± 1.0	98.7 ± 1.2
10 min_Anesthesia	98.1 ± 1.1	98.4 ± 1.1	98.6 ± 1.2	98.6 ± 1.1
30 min_Anesthesia	98.5 ± 1.2	98.6 ± 1.0	98.7 ± 1.2	98.7 ± 1.0
60 min_Anesthesia	98.4 ± 1.0	98.8 ± 1.1	98.7 ± 1.1	98.8 ± 1.1
End of operation	98.3 ± 1.3	98.8 ± 1.1	98.8 ± 1.0	98.8 ± 1.1
Cardiac output (CO) (L/min)				
Baseline	6.99 ± 1.12	6.98 ± 1.16	7.07 ± 1.12	7.08 ± 1.12
10 min_Anesthesia	4.44 ± 0.84^a^	6.06 ± 1.13^c^	6.21 ± 1.17^c^	6.44 ± 1.24^c^
30 min_Anesthesia	4.68 ± 1.00^a^	6.31 ± 1.30^c^	6.30 ± 1.31^c^	6.47 ± 1.33^c^
60 min_Anesthesia	4.93 ± 1.05^a^	6.48 ± 1.35^c^	6.52. ± 1.3^c^	9.04 ± 1.81^acef^
End of operation	4.96 ± 1.08^a^	6.78 ± 1.34^c^	6.78 ± 1.39^c^	9.41 ± 1.91^acef^
Systolic volume (SV) (mL/beat)				
Baseline	85.3 ± 5.0	85.3 ± 5.4	85.4 ± 5.2	85.6 ± 5.1
10 min_Anesthesia	65.3. ± 6.4^a^	91.2 ± 7.1^c^	92.5 ± 7.3^c^	92.7 ± 7.4^c^
30 min_Anesthesia	66.4 ± 6.5^a^	93.4 ± 7.3^c^	93.6 ± 7.2^c^	94.2 ± 7.5^c^
60 min_Anesthesia	68.3 ± 6.7^a^	93.6 ± 7.5^c^	93.8 ± 7.3^c^	125.2 ± 9.7^acef^
End of operation	68.6 ± 6.9^a^	93.9 ± 7.6^c^	94.1 ± 7.8^c^	130.2 ± 10.1^acef^

Data are presented as mean ± standard deviation (SD)

^a^ *P* < 0.05, compared with that at 10 min before anesthesia induction

^c^ *P* < 0.05, compared with the control group (C)

^e^ *P* < 0.05, compared with the M1 group

^f^ *P* < 0.05, compared with the M2 group

Table 3 summarizes the incidences of cardiovascular complications during surgery in the different groups. Compared with the control group, all methoxamine groups had a lower incidence and shorter duration of low blood pressure, whereas the M3 group had a higher incidence of high blood pressure (*P* < 0.01 or <0.05).

**Table 3:** Incidences of cardiovascular complications and duration of low blood pressure (LBP) during surgery in different groups

Group	HBP	LBP	Tachycardia	Bradycardia	Pulmonary edema	Accumulated duration of LBP, min
	*n* (%)					
C	0 (0)	16 (21.3)	0 (0)	19 (25.3)	2 (2.7)	2.3 ± 0.5
M1	0 (0)	0 (0)^a^	0 (0)	16 (21.3)	0 (0)^b^	0^b^
M2	0 (0)	0 (0)^a^	0 (0)	18 (24.0)	0 (0)^b^	0^b^
M3	32 (42.6)^a^	0 (0)^a^	0 (0)	17 (22.7)	0 (0)^b^	0^b^

*HBP* high blood pressure

^a^ *P* < 0.01

^b^ *P* < 0.05, compared with the control group (C)

Table 4 summarizes the intraoperative liquid transfusion volume, perioperative urine volume, and drug usage in the four groups. Compared with the control group, all methoxamine treatment groups had significantly lower intraoperative liquid transfusion volume, less ephedrine usage, higher intraoperative urine volume, and lower postoperative 24 h urine volume (*P* < 0.01 or <0.05). No statistical differences in ropivacaine and sufentanil usage were observed among the four groups (*P* > 0.05)

**Table 4:** Intraoperative transfusion volume, perioperative urine volume, and usage of analgesics in four groups (*n* = 75 per group)

Group	The total volume of liquid infused, mL	Intraoperative ephedrine dose, mg	Intraoperative ropivacaine dose, mg	Intraoperative urine output, mL	24-h postoperative urine output, mL	Postoperative ropivacaine dose, mg	Postoperative sufentanyl dose, mg
C	2447.7 ± 175.3	47.5 ± 6.7	121.3 ± 41.4	239.2 ± 73.4	2128.5 ± 136.7	195.4 ± 3.4	29.31 ± 0.51
M1	1390.7 ± 286.5^b^	0^a^	117.4 ± 38.2	597.6 ± 121.5^b^	1339.4 ± 171.2^b^	196.0 ± 4.0	29.40 ± 0.60
M2	1385.9 ± 283.4^b^	0^a^	122.1 ± 40.5	602.4 ± 119.7^b^	1340.8 ± 172.7^b^	194.9 ± 3.1	29.25 ± 0.45
M3	1379.2 ± 285.8^b^	0 (0)^a^	118.6 ± 39.3	592.5 ± 117.1^b^	1336.1 ± 167.9^b^	194.9 ± 3.1	29.25 ± 0.45

Data are presented as mean ± SD

^a^ *P* < 0.01

^b^ *P* < 0.05, compared with the control group (C)

Table 5 shows the results of assessments of analgesia quality and comfort level using the VAS and BCS, respectively. Satisfactory analgesia was achieved at all time points in all groups, with no statistical differences observed (*P* > 0.05).

**Table 5:** Assessment of analgesia quality and comfort level at multiple postoperative time points in four groups (*n* = 75 per group)

	Group	1 h	6 h	12 h	18 h	24 h	36 h	48 h
VAS	C	1.75 ± 0.13	1.77 ± 0.13	1.77 ± 0.12	1.76 ± 0.13	1.75 ± 0.12	1.76 ± 0.12	1.77 ± 0.11
	M1	1.76 ± 0.12	1.78 ± 0.14	1.76 ± 0.13	1.77 ± 0.14	1.76 ± 0.13	1.77 ± 0.11	1.78 ± 0.12
	M2	1.75 ± 0.12	1.77 ± 0.13	1.78 ± 0.11	1.77 ± 0.12	1.76 ± 0.14	1.77 ± 0.13	1.78 ± 0.14
	M3	1.77 ± 0.11	1.76 ± 0.12	1.76 ± 0.13	1.76 ± 0.14	1.75 ± 0.13	1.76 ± 0.11	1.78 ± 0.11
BCS	C	3.88 ± 0.10	3.86 ± 0.12	3.87 ± 0.09	3.87 ± 0.07	3.87 ± 0.08	3.89 ± 0.10	3.88 ± 0.07
	M1	3.86 ± 0.12	3.85 ± 0.13	3.87 ± 0.12	3.86 ± 0.10	3.86 ± 0.11	3.88 ± 0.11	3.89 ± 0.10
	M2	3.87 ± 0.13	3.87 ± 0.10	3.86 ± 0.10	3.87 ± 0.10	3.87 ± 0.08	3.87 ± 0.10	3.88 ± 0.09
	M3	3.87 ± 0.11	3.86 ± 0.13	3.86 ± 0.11	3.86 ± 0.12	3.87 ± 0.12	3.88 ± 0.11	3.88 ± 0.10

Data are presented as mean ± SD. No statistical difference was observed

As shown in Table 6, MMSE test scores were lower at 1 and 3 days after operation in the M3 group than in the control group and were lower in both of these groups than those at 1 day before surgery (*P* < 0.05), with the M3 group showing a lower mean score than the control group (*P* < 0.05). Meanwhile, the incidence of POCD was remarkably higher in the M3 group (18.7%) than in the control (5.3%), M1 (6.7%), and M2 (6.7%) groups (*P* < 0.05), compared with that at 10 min before anesthesia (baseline; *P* < 0.05). As shown in Table 7, blood TNF-α levels at the end of surgery and 1 and 2 days after surgery were higher in the M3 group than in the control, M1, and M2 groups and were higher than the respective baseline levels in all groups (*P* < 0.05).

**Table 6:** MMSE test scores in four groups at different time points (*n* = 75 per group)

Group	1 d before operation	1 d post operation	3 d post operation	7 d post operation	POCD, *n* (%)
C	27.8 ± 2.1	24.3 ± 2.5^a^	25.2 ± 2.4 ^a^	27.6 ± 2.2	4 (5.3)
M1	27.7 ± 2.1	24.5 ± 2.4^ac^	24.9 ± 2.5^a^	27.5 ± 2.2	5 (6.7)
M2	27.9 ± 2.1	25.1 ± 2.5^ac^	25.3 ± 2.3^a^	27.6 ± 2.3	5 (6.7)
M3	27.8 ± 2.0	22.2 ± 2.4^ac^	22.8 ± 2.5^ac^	27.4 ± 2.0	14 (18.7)^c^

Data are presented as mean ± SD

^a^ *P* < 0.05, compared with 1 day before operation

^c^ *P* < 0.05, compared with the control group

**Table 7:** Serum TNF-α levels (pg/mL) in four groups at different time points (*n* = 75 per group)

Group	Baseline	End of operation	24 h after operation	48 h after operation
C	12.64 ± 3.21	31.95 ± 6.19^a^	32.12 ± 6.59^a^	35.09 ± 6.21^a^
M1	13.09 ± 3.14	32.09 ± 6.20^a^	33.17 ± 7.35^a^	36.11 ± 6.23^a^
M2	12.08 ± 3.22	34.15 ± 6.25^a^	36.19 ± 6.54^a^	39.15 ± 7.17^a^
M3	12.11 ± 3.19	58.08 ± 6.21^abcd^	61.7 ± 13.31^abcd^	64.14 ± 14.19^abcd^

Data are presented as mean ± SD

^a^ *P* < 0.05, compared with that at baseline

^b^ *P* < 0.05, compared with the M1 group

^c^ *P* < 0.05, compared with the control group

^d^ *P* < 0.05, compared with the M2 group

## Discussion

In this study, at all studying time points during anesthesia, the M1 and M2 groups only showed decreases in the CVP and HR, whereas the control group exhibited significant decreases in various hemodynamic parameters. Meanwhile, compared with the control group, all methoxamine treatment groups required less transfusion volume, showed increased urine excretion during surgery and decreased urine excretion after surgery, suggesting less urine retention. On the other hand, as the surgical time increased, intravenous infusion of methoxamine at 4 μg·kg^−1^·min^−1^ caused increases in blood pressure (in up to 42.6% of the 75 patients), cardiac output, and systolic stroke volume, leading to increased myocardium oxygen consumption. These results demonstrated that intravenous infusion of methoxamine at an adequate dosage (2–3 μg·kg^−1^·min^−1^) could provide better control of the hemodynamics during epidural anesthesia and surgery. The decreases in CVP in all groups were mainly due to dilation of peripheral resistance vessels and venous vessels, caused by sympathetic block associated with epidural anesthesia. The high incidence of bradycardia in all groups may be the result of increased activity of the vagus nerve due to epidural anesthesia, which on the other hand further confirms that methoxamine has the potential to regulate vagus nerve activity via the carotid sinus baroreflex, reducing myocardium oxygen consumption while maintaining a high blood pressure [17, 18]. The concomitant low blood pressure (in 21.3% of the 75 patients) and pulmonary edema (2.7%) may be caused by the dilation of peripheral vessels and excessive liquid transfusion in efforts to maintain a normal blood volume.

Surgical insults can activate the peripheral immune system and cause inflammation, leading to the excretion of various inflammatory cytokines such as interleukin-1β, interleukin-6, and TNF-α, which can be transported through the blood–brain barrier and activate astrocytes and microglia to induce the cytotoxic reaction and damage cognitive function [9, 10, 25, 26]. In elderly rats with POCD, microglia and neuronal dendrites were observed to be significantly activated in the hippocampus, accompanied by dramatic increases of TNF-α and IL-1β levels, suggesting that POCD probably originates from neuroinflammation in the hippocampus [27]. In the present study, all groups showed higher TNF-α levels after surgery compared with baseline levels, and the M3 group had a higher TNF-α level than the control, M1, and M2 groups. Meanwhile, the incidence of POCD was observed to be much higher in the M3 group (18.7%) than in the control (5.3%), M1 (6.7%), and M2 (6.7%) groups. These results support that POCD can be associated with excessive excretion of TNF-α, consistent with the findings reported by the above-described literature. In the control group, the incidence of POCD was relatively low, though 21.3% of the patients presented with low blood pressure. This was possibly because the accumulative duration of low blood pressure was actually short in these patients (2.3 ± 0.5 min) due to timely intervention.

Benzodiazepines, anticholinergic agents, pain, and hypoxemia also can increase the risk of developing POCD [28–31]. In this study, the usage of benzodiazepines and anticholinergic agents were avoided in all groups both before and after surgery. The intraoperative CVP and SpO_2_ were in the normal ranges in all patients. Satisfactory analgesia was achieved in all patients. These facts also to some extent helped to reduce the potential risk of developing POCD.

The major limitation of this study is that as compared with patients who received lower doses, the patients who received methoxamine at the dose of 4 μg·kg^−1^·min^−1^ did not show further improved intraoperative hemodynamic stability. Furthermore, these patients underwent elevated myocardial oxygen consumption and showed higher incidence of POCD. Future studies should include compensation measures for unexpected loss in the patients.

## Conclusion

In conclusion, intravenous infusion of methoxamine at 2–3 μg·kg^−1^·min^−1^ was demonstrated effective in maintaining stable hemodynamics in elderly patients during epidural anesthesia for hip-joint replacement surgery, without increasing the incidence of POCD.

## Abbreviations

ASA: American Society of Anesthesiologists
MMSE: Mini-mental state examination
POCD: Postoperative cognitive dysfunction
TNF-α: Tumor necrosis factor-α
